# Humanized CD19 CAR-T cells in relapsed/refractory B-ALL patients who relapsed after or failed murine CD19 CAR-T therapy

**DOI:** 10.1186/s12885-022-09489-1

**Published:** 2022-04-12

**Authors:** Lihong An, Yuehui Lin, Biping Deng, Zhichao Yin, Defeng Zhao, Zhuojun Ling, Tong Wu, Yongqiang Zhao, Alex H. Chang, Chunrong Tong, Shuangyou Liu

**Affiliations:** 1 Department of Hematology, Beijing Boren Hospital, No.6 South Zhengwangfen, Fengtai District, Beijing, 100070 China; 2 Cytology Laboratory, Beijing Boren Hospital, Beijing, China; 3 Department of Hematopoietic Cell Transplantation, Beijing Boren Hospital, Beijing, China; 4 Clinical Translational Research Center, Shanghai Pulmonary Hospital, Tongji University School of Medicine, Shanghai, China

**Keywords:** Chimeric antigen receptor-T, Acute lymphoblastic leukemia, Relapsed/refractory, Single-chain variable fragment

## Abstract

**Background:**

For CD19-positive relapsed/refractory B-cell acute lymphoblastic leukemia (r/r B-ALL) after treatment with murine CD19 (mCD19) CAR-T, the reinfusion of mCD19 CAR-T cells may be ineffective due to anti-mouse single-chain variable fragment (scFv) antibody caused by mCD19 CAR. To overcome this immunogenicity, we applied humanized CD19 (hCD19) CAR-T cells to treat r/r B-ALL patients with prior mCD19 CAR-T therapy.

**Methods:**

Nineteen pediatric and adult patients were included, 16 relapsed after and 3 were primarily resistant to mCD19 CAR-T. All patients presented with more than 5% blasts in bone marrow and/or extramedullary disease, and still showed CD19 antigen expression. Humanized CD19-CARs were lentiviral vectors carrying a second generation CAR with 4–1-BB co-stimulatory and CD3ζ signaling domains. Patient-derived cells were collected for producing CAR-T cells, the median dose of infused hCD19 CAR-T cells was 2.4 × 10^5^/kg (range, 1.0–18.0 × 10^5^/kg).

**Results:**

hCD19 CAR-T resulted in a complete remission (CR) rate of 68% (13/19). Among 13 remission patients, 11 underwent allogeneic hematopoietic cell transplantation (allo-HCT) (3 were second HCT) and 10 remained in CR; the event-free survival rates at 12–18 months were 91% in 11 patients received following allo-HCT and 69% in all CR patients. Six cases had no response to hCD19 CAR-T, 3 died of disease progression; another 3 received salvage second transplantation, of them, 2 relapsed again (one died). Cytokine release syndrome (CRS) occurred in 95% (18/19) of patients, most CRS events were grade 1 and grade 2 (*n* = 17), there was only one grade 4 CRS. Two cases experienced grade 1 neurotoxicity.

**Conclusions:**

Humanized CD19 CAR-T cell therapy could be a treatment option for CD19-positive B-ALL patients who relapsed after or resisted prior murine CD19 CAR-T, hCD19 CAR-T followed by allo-HCT provided a longer remission in CR patients. Nevertheless, the prognosis of non-responders to hCD19 CAR-T remained dismal.

**Trial registration:**

Chinese Clinical Trial Registry/WHO International Clinical Trial Registry (ChiCTR1900024456, URL: www.chictr.org.cn); registered on July 12, 2019.

**Supplementary Information:**

The online version contains supplementary material available at 10.1186/s12885-022-09489-1.

## Introduction

Although CD19-specific chimeric antigen receptor (CAR) T-cell therapy has achieved high complete remission (CR) rates in relapsed/refractory B-cell acute lymphoblastic leukemia (r/r B-ALL) [1–4], a minority of patients have no response and many patients relapse with either CD19 positivity or CD19 negativity. For patients with CD19 positivity relapsed after or resisted to prior CD19 CAR-T therapy, retreatment with CD19 CAR-T cells remains a treatment option. At present, most of CD19 CAR-T cell products are murine-derived, the reinfusion of same murine CD19 (mCD19) CAR-T cells may not be effective owing to anti-mouse single-chain variable fragment (scFv) antibody caused by mCD19 CAR [5–7], whereas humanized CD19 (hCD19) CAR may overcome this immunogenicity.

It has been reported that one patient relapsing after mCD19 CAR-T did not respond to a secondary infusion of mCD19 CAR-T cells but obtained CR following hCD19 CAR-T [8]; and 4 of 5 patients who had received previous mCD19 CAR-T and relapsed with CD19^+^ B lymphoblastic cells achieved CR by treatment with humanized selective CD19 CAR-T [9]. Very recently, while this paper was in preparation, a new study showed that, in 33 childhood and young adult B-ALL patients with prior mCD19 CAR exposure (CD19+ relapse, *n* = 15; B-cell recovery, *n* = 16; no response to prior CAR-T cells, *n* = 2), the overall response rate at 1 month after humanized CD19 CAR-T cell infusion was 64% [10].

In this study, we used hCD19 CAR-T cells to treat 19 relapsed/refractory B-ALL patients who previously received mCD19 CAR-T but still had a high level of CD19 antigen expression, including 16 cases relapsed after and 3 cases primarily failed to mCD19 CAR-T, the treatment response at 1 month and follow-up outcomes were evaluated.

## Patients and methods

### Patients

**Fig. 1:**
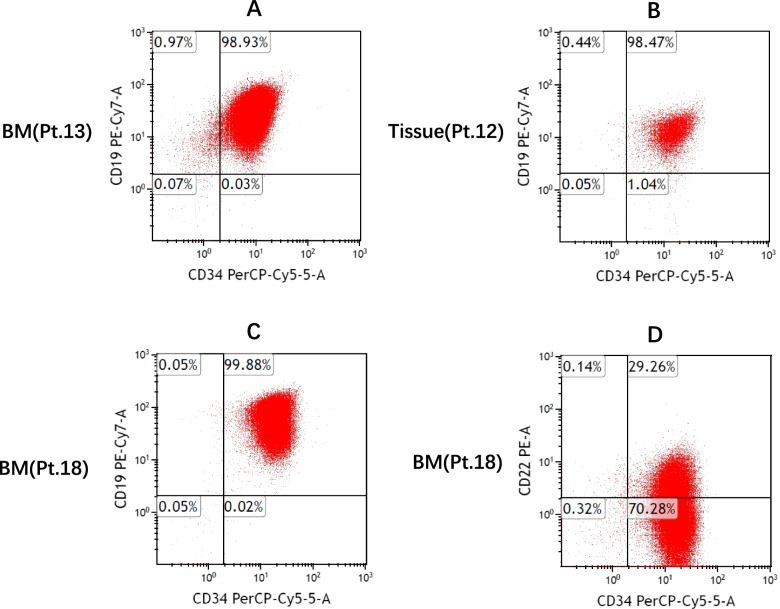
CD19/CD22 antigen expression identified by flow cytometry. **A**, **B**, **C** Representatives of CD19 expression on lymphoblasts before hCD19 CAR-T therapy in 3 patients (Pt.). Samples were from bone marrow (BM) or tissue (Pt.12 relapsed with extramedullary disease only and without BM involvement). **D** Partial CD22 expression in Pt.18 who failed two mCD19 CAR-T therapies

From January 2019 to November 2020, a total of 21 pediatric and adult patients with relapsed/refractory B-ALL were enrolled (7 were in the proof of concept procedure before trial registration date), cases only with minimal residual disease (MRD) were not included. All patients had a history of prior murine CD19 CAR-T (with or without CD22 CAR-T) therapy, and their leukemia cells expressed high levels of CD19 antigen (≥95% of blasts were positive for CD19) identified by multiparameter flow cytometer (FCM) (Fig. 1A-C). Patients with leukemia cells in cerebrospinal fluid but without intracranial lesions were eligible. Eastern Cooperative Oncology Group performance status (ECOG PS) of 0–2 and adequate organ function were required. The details of inclusion and exclusion criteria were showed in supplemental method. Among 21 enrolled patients, two were excluded: one had received CD79b and CD20 CAR-T cells except for CD19 and CD22 CAR-T, and another one once received humanized CD19 CAR-T; the remaining 19 patients underwent hCD19 CAR-T therapy. The last cell infusion was performed in November 2020, and the cut-off date of follow-up was as of July 31, 2021.

### Humanized CD19 CAR-T cells and treatment

Humanized CD19 CARs were lentiviral vectors carrying a second generation CAR with 4–1-BB co-stimulatory and CD3ζ signaling domains (from Shanghai YaKe Biotechnology Ltd., Shanghai, China). The antigen recognition domains of CD19-specific CARs are single-chain variable fragments obtained from a human antibody phage display library. Patient-derived cells were collected for producing CAR-T cells, which were transfected by lentiviral vectors and cultured for 5–8 days. The dosage of infused CAR-T cells was set at ≤5 × 10^5^/kg of body weight for post-transplantation patients and ≤ 50 × 10^5^/kg for pre-transplantation patients, usually between 1 and 10 × 10^5^/kg of body weight. The exact dosage for each patient depended on the cell product, patient’s situation and physician’s decision. Patients received lymphodepleting agent fludarabine (30 mg/m^2^/day) with or without cyclophosphamide (250 mg/m^2^/day) for 3 days prior to cell infusion, additional short-term and non-intensive chemo drugs before or together with lymphodepleting agents were allowed for these heavily treated patients. Targeted drugs such as tyrosine kinase inhibitors (TKIs) and intracranial chemotherapy could be used. All medications were withdrawn at least 1–2 days before CAR-T cell infusion.

This phase I study was approved by Beijing Boren Hospital ethics committee and registered on Chinese Clinical Trial Registry/WHO International Clinical Trial Registry (ClinicalTrials#: ChiCTR1900024456), written informed consents were obtained in accordance with the Declaration of Helsinki, for children under 18 years old, the participant consent form was signed by their parent/legal guardian.

### Assessments

Disease status and treatment effects were determined according to the guidelines of the National Comprehensive Cancer Network (NCCN) [11]. Immunophenotype and MRD were performed by flow cytometry on BD FACSCanto II or FACSCalibur, monoclonal antibodies were purchased from BD pharmingen (San Diego, California). Fusion genes were assayed by real-time quantitative polymerase chain reaction (qPCR) (Applied Biosystems 7500, Thermo Fisher Scientific). The MRD level below 1 × 10^− 4^ (both FCM and qPCR) was defined as negative. The extramedullary disease (EMD) was evaluated by PET-CT/CT/ultrasound. Cytokine release syndrome (CRS) and neurotoxicity were graded by the ASTCT grading system [12]. CAR-T cell numbers were determined through flow cytometric quantitation, FITC-conjugated CD19-CAR detection reagents were provided by Shanghai YaKe Biotechnology Ltd. (Shanghai, China). B-cell aplasia (BCA) was defined as less than 3% CD19 positive lymphocytes in bone marrow (BM). Cytokines were assayed by enzyme-linked immunosorbent assay (R&D Systems, Bio-Techne). Gene mutations were detected by targeted high-throughput sequencing (Illumina NextSeq 550).

### Statistics

R software version 4.1.1 was used to graph and analyze data. Categorical variables were compared by the Fisher exact test. The probability of event-free survival (EFS) was estimated by the Kaplan–Meier method, the time-to-event (death, relapse or survival) analyses were calculated from the date of hCD19 CAR-T cell infusion to the date of death, relapse or last follow-up.

## Results

### Patient characteristics

A total of 19 r/r B-ALL patients were eligible for hCD19 CAR-T cell infusion, 16 relapsed after mCD19 CAR-T (one failed the second infusion of mCD19 CAR-T cells) and 3 were primarily resistant to mCD19 CAR-T; the median age was 20 (range, 4–49) years, consisting of 11 adults and 8 children younger than 18 years old. Two cases had BCR-ABL fusion gene and 7 were Ph-like B-ALL (2 simultaneously harbored *TP53* gene mutations). Twelve patients (63%, 12/19) showed bone marrow relapse and blast cells in BM varied between 7 and 98%; 7 cases (37%, 7/19) relapsed with extramedullary disease (EMD only, *n* = 1; both EMD and BM, *n* = 6), of them, 2 had central nervous system leukemia (CNSL) and 5 showed multifocal diseases (defined as ≥ two sites of EMD) including one with CNSL. Ten cases underwent allogeneic hematopoietic cell transplantation (allo-HCT) with no graft-versus-host disease (GVHD) at enrollment (Table 1 and Table S1) .

**Table 1:** Patient characteristics

Characteristics	No.(***n*** = 19)	% of patients
**Age (years)**		
Median	20 (range 4–49)	
Children (< 18)	8	42
Adults	11	58
**Sex**		
Male	10	53
Female	9	47
**Prior allo-HCT**		
Yes	10	53
No	9	47
**CD22 CAR-T (simultaneously with or after mCD19 CAR-T)**		
Yes	11	58
No	8	42
**Disease status at enrollment**		
Isolated BM (blasts 7–98%)	12	63
Extramedullary disease (EMD)	7	37
CNS	2	
Multiple sites (1 with CNS)	5	
**Adverse genetic changes**		
BCR/ABL	2	11
Ph-like (2 with *TP53* gene mutation)	7	37
**Interval time between last mCD19**		
**CAR-T and hCD19 CAR-T (months)**		
< 6	6	32
6–12	4	21
> 12	9	47

*Abbreviations*: *No*. Number, *allo-HCT* Allogeneic hematopoietic cell transplantation, *BM* Bone marrow, *CNS* Central nervous system, *mCD19* Murine CD19, *hCD19* Humanized CD19

All 19 patients previously received mCD19 CAR-T cells, 16 received one infusion and 3 received two infusions. In 3 cases treated with second mCD19 CAR-T (2 relapsed after and 1 failed first mCD19 CAR-T), 1 obtained CR again and 2 were non-responders (patient #18 was resistant to both first and second mCD19 CAR-T). Although patient #18 failed two mCD19 CAR-T therapies, the preferred target antigen was still CD19 instead of CD22 because her blast cells expressed a high level of CD19 but a low level of CD22 (Fig. 1C-D), moreover, we sequenced her CD19 gene and potential gene mutations were excluded. Eleven cases also received CD22 CAR-T treatment which was followed by (*n* = 8) or simultaneously with mCD19 CAR-T (*n* = 3) (Table 1 and Table S1).

### Humanized CD19 CAR-T cell expansion and toxicity

The median dose of infused hCD19 CAR-T cells was 2.4 × 10^5^/kg (range, 1.0–18.0 × 10^5^/kg). CAR-T cell expansion was seen in 17 patients, the median peak number of CAR-T cells in peripheral blood was 5.2 × 10^6^/L assayed by FCM (Table S2 and Fig. S1), which was lower than that (72 × 10^6^/L) in our patients who firstly received mCD19 CAR-T [13]. Additionally, CAR-T cells could not be detected in 9 patients (53%, 9/17) within 10–37 days after cell infusion. These data implied that, in this cohort of patients having received repeated CAR-T cell infusions (3 with two CD19 and 11 also with CD22 CAR-T cell infusion), CAR-T cells could not proliferate at a high level and persist a longer time. In two patients with no detectable CAR-T cells, one (patient #16) presented grade 1 CRS, significantly increased cytokines (Table S3) and obtained CR, furthermore, he had no CD19-positive B-cells in BM after CAR-T, we assumed that this case actually had expanded CAR-T cells whereas these cells could not be detected by FCM (no PCR data) due to an uncertain reason. Cytokine release syndrome occurred in 95% (18/19) of patients, most CRS events were grade I (*n* = 15) and grade 2 (*n* = 2), there was only one grade 4 CRS. Cytokine release syndrome was managed with corticosteroids according to the approach we described in previous work [14], 3 patients were given additional plasmapheresis. Grade I neurotoxicity occurred in 2 cases. There was no treatment-related death.

### Treatment response

On day 30 after hCD19 CAR-T cell infusion, 13 (68%, 13/19) patients achieved complete remission (including CR with incomplete blood count recovery) and 10 were MRD-negative (2 cases with multifocal EMDs could not be evaluated MRD and 1 had no MRD data). There was no significant difference in response rates between patients with or without prior CD22 CAR-T therapy (7/11, 64% vs. 6/8, 75%, *p* = 1.0), and patients with < 12-month or > 12-month duration from last mCD19 CAR-T to hCD19 CAR-T (8/10, 80% vs. 5/9, 56%, *p* = 0.3498). Among 13 CR patients, 10 relapsed after mCD19 CAR-T (patient #2 failed the second reinfusion of mCD19 CAR-T cells) and 3 were primarily resistant to mCD19 CAR-T (patient #18 failed both first and second mCD19 CAR-T). It was surprising that all 3 cases who primarily resisted mCD19 CAR-T obtained CR.

Six patients (32%, 6/19) had no response (NR) to hCD19 CAR-T therapy. Although 5 non-responders showed CAR-T cell proliferation, the leukemic cells of four patients and the normal B-cells of one patient who had no BM involvement (EMD only) still expressed CD19 antigen after hCD19 CAR-T, these results revealed that, among these 5 patients, the expanded CAR-T cells did not target and kill CD19-positive B-cells. This phenomenon was reported by another group in which CTL019 cells proliferated in vivo and were detectable in the blood and bone marrow of patients who had a response and patients who did not have a response [15]. All 6 non-responders were post-HCT patients, the CR rate was 40% (4/10) in post-HCT subgroup and 100% (9/9) in pre-HCT subgroup (*p* = 0.0108), indicating that hCD19 CAR-T therapy was more beneficial to pre-HCT cases among this cohort of patients.

### Follow-up

**Fig. 2:**
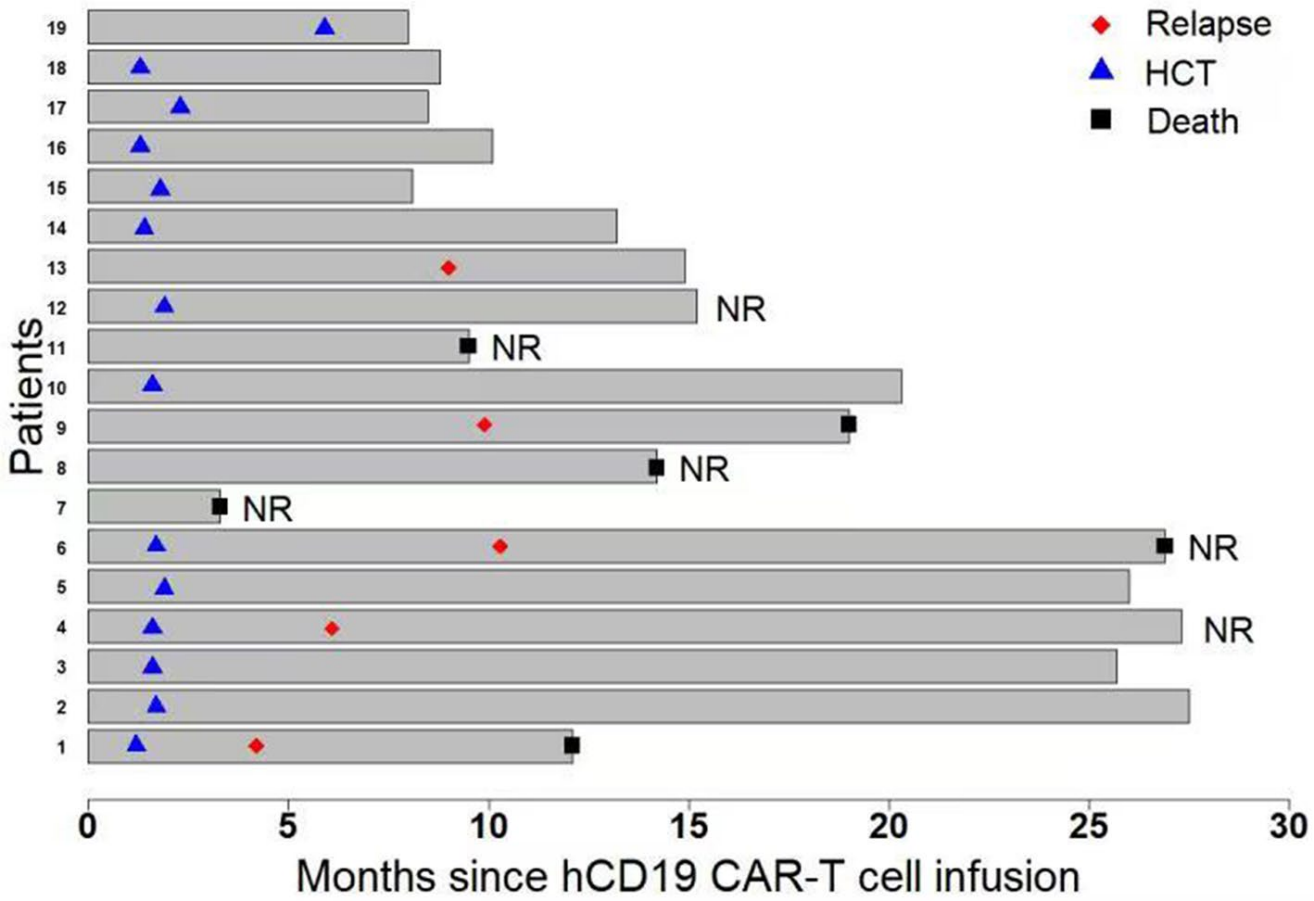
The follow-up information of each patient. The length of each bar represents a patient’s survival time (months) from the date of hCD19 CAR-T cell infusion to the date of death or last follow-up. Among 6 non-responders to hCD19 CAR-T, 3 died of disease progression; 3 received salvage transplantation and achieved remission, of them, 2 relapsed again and 1 died

**Fig. 3:**
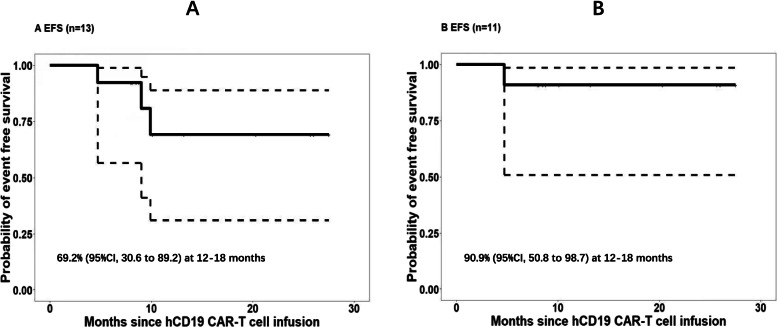
Kaplan-Meier analysis on event-free survival (EFS) in complete remission (CR) patients. **A** EFS in all 13 CR patients. **B** EFS in 11 patients who received allogeneic hematopoietic cell transplantation following hCD19 CAR-T

The follow-up information of patients was shown in Fig. 2 and Table 2. Among 13 remission patients, 11 underwent allo-HCT (3 were second HCT) in 6 months (within 1.3–2.3 months, *n* = 10; at 5.9-month, n = 1) after hCD19 CAR-T. At a median follow-up of 12.1 (range, 8–27.5) months, 10 patients remained in CR, and only one relapsed at 3 months and died at 10.4 months since allo-HCT; there was no transplantation-related mortality. Another 2 CR patients refused transplantation, both relapsed at 9.9 and 9 months with CD19 positivity (their B-cell recovery appeared at 1.2 and 3.4 months, respectively) post hCD19 CAR-T, as of the last follow-up, 1 died and 1 was alive. The Kaplan–Meier analysis revealed that the EFS rates at both 12 and 18 months were 91% (95% confidence interval [CI], 50.8 to 98.7) in 11 patients received allo-HCT following hCD19 CAR-T and 69% (95%CI, 30.6 to 89.2) in all 13 CR patients including 2 without further HCT (Fig. 3).

**Table 2:** hCD19 CAR-T treatment and outcomes

Variables	No. (***n*** = 19)	% of patients	Numbers of patients		
			Total	CR	Relapse/Death
**Grade of CRS**					
0	1	5	–	–	–
1	15	79	–	–	–
2	2	11	–	–	–
4	1	5	–	–	–
**Neurotoxicity**					
No	17	89	–	–	–
Grade 1	2	11	–	–	–
**Treatment response on D30**					
CR	13	68	–	–	–
NR	6	32	–	–	–
**Follow-up**					
**CR group**			13		
allo-HCT	–	–	11	10	1/1(same case)
non-HCT	–	–	2	0	2/1
**NR group**			6		
allo-HCT	–	–	3	1	2/1
non-HCT	–	–	3	0	0/3

*Abbreviations*: *CRS* Cytokine release syndrome, *CR* Complete remission, including CR with incomplete blood count recovery, *NR* No response, *allo-HCT* Allogeneic hematopoietic cell transplantation

Among 6 NR patients, 3 died of disease progression and 1 of them failed subsequent CD22 CAR-T therapy as well. Another 3 cases received salvage transplantation (all were second allo-HCT) and achieved CR, 1 had been in CR status for 13.3 months and 2 relapsed again at 4.5 and 8.6 months since allo-HCT, in 2 relapsed patients, one died and one was alive by receiving other ant-cancer therapies.

In our previous study regarding CAR-T therapy for post-HCT B-ALL patients, CAR-T associated GVHD occurred in 6 (all were CR patients) of 26 patients, 2 cases developed acute GVHD and 4 with chronic GVHD (cGVHD) before CAR-T showed persistent or worsened pre-existing cGVHD after CAR-T cell infusion [13]. Here, in 10 post-HCT patients, GVHD was not observed after hCD19 CAR-T, the possibilities of that there was no CAR-T associated GVHD could be: 1) these 10 patients did not have pre-existing cGVHD; 2) 6 cases had no response to hCD19 CAR-T and therefore their donor T-cells causing GVHD were not increased and activated; 3) 3 of 4 CR patients underwent second HCT immediately following hCD19 CAR-T, whose GVHD related to CAR-T could not be followed-up.

## Discussion

With the broad use of CD19 CAR-T cells in B-cell lymphoblastic leukemia, post-CAR-T relapse and CAR-T resistance emerge as the new clinical problems [16, 17]. To date, most of CD19 CAR-T products including those approved by U.S. Food and Drug Administration are murine-derived. It has been reported that the specific IgA to murine scFv of FMC63 could be detected in sera of B-ALL patients received mCD19 CAR-T, and the existence of murine CAR-specific IgA may render the second mCD19 CAR-T treatment ineffective [9].

To overcome the treatment failure caused by immunogenicity of scFv from murine CD19 CAR in patients with prior mCD19 CAR-T exposure, we applied humanized CD19 CAR-T cells to treat r/r B-ALL patients who relapsed after or had no response to mCD19 CAR-T but still showed high levels of CD19 expression. A total of 19 pediatric and adult patients were included, more than half of them (58%, 11/19) had received CD22 CAR-T therapy as well. Humanized CD19 CAR-T cells resulted in a CR rate of 68% (13/19). Although this CR rate was lower than those obtained in patients firstly accepted mCD19 CAR-T in which CR rates reached 81–90% [1–4], the therapeutic efficiency of hCD19 CAR-T cells for these heavily treated patients who had no more treatment options was encouraging. A recent study also reported that the humanized CD19 CAR-T produced initial responses in 64% of patients treated for CD19+ relapse, early B-cell recovery, or nonresponse after prior murine CAR-T cells [10].

One patient who relapsed from first mCD19 CAR-T and failed second mCD19 CAR-T obtained complete remission after hCD19 CAR-T therapy, which verified that hCD19 CAR-T could overcome the treatment failure of reinfusion of mCD19 CAR-T cells. It was interesting that all three patients primarily resistant to mCD19 CAR-T (one failed two mCD19 CAR-T therapies) obtained CR following the subsequent hCD19 CAR-T. It remains unknow why these cases having no response to mCD19 CAR-T achieved CR by hCD19 CAR-T, their previous mCD19 CAR-T therapies were performed in other hospitals with no treatment details, we assumed that the failure of mCD19 CAR-T therapy might be related to the T-cell function at cell collection, the procedure of CAR-T cell manufacturing or different clinical situations.

Among 13 CR patients, 11 underwent allo-HCT (3 were second HCT) after hCD19 CAR-T and 10 remained in remission (1 relapsed and died); whereas another 2 cases refused following HCT relapsed again in 10 months post hCD19 CAR-T (1 died). The EFS rates at 12–18 months were 91% in 11 patients received further allo-HCT and 69% in all 13 CR patients. These results demonstrated that allo-HCT even second allo-HCT could provide a longer event-free survival for CR patients, therefore, allo-HCT following hCD19 CAR-T is suggested as a potent consolidation for these patients. We noted that there was a higher EFS rate here in post-HCT patients, which could be explained by the fact that this was a small group of patients and there was no transplantation-related mortality. Among 6 non-responders to hCD19 CAR-T, 3 cases received the salvage second transplantation and achieved CR, however, 2 of them relapsed again and 1 died. The different consequences of transplantation between patients with or without remission before allo-HCT supported the previous conclusion of that the CR status at the time of HCT was the most favorable factor of successful transplantation [18, 19], regardless of first or second allo-HCT. Another 3 NR patients without undergoing HCT died of disease progression. The follow-up outcomes of the patients who failed hCD19 CAR-T therapy revealed that they had a very poor prognosis, the salvage transplantation could not maintain a sustained event-free remission either.

CD22 CAR-T therapy also showed a promising treatment efficiency in r/r B-ALL patients including those relapsed after mCD19 CAR-T especially with CD19 negativity [20, 21]. In this cohort of patients, more than half of them (58%, 11/19) had received CD22 CAR-T cell infusion before enrollment, either as a treatment (*n* = 5) or as a consolidation following CD19 CAR-T (*n* = 6). Of all 19 patients, 2 presented partial CD22 expression (20–80% of blasts were CD22-positive) and 17 had normal CD22 expression (> 80% of blasts were CD22-positive), the latter 17 patients therefore could be treated by both CD19 and CD22 CAR-T cells. Considering that these patients had a high level of CD19 expression (≥95% of blasts were CD19-positive) and CD22 CAR-T therapy showed a little lower CR rates (73–80%) [20, 21] compared to CD19 CAR-T, we preferred to choose CD19 CAR-T as a targeted treatment. Among 6 patients having no response to hCD19 CAR-T, one was subsequently infused with CD22 CAR-T cells and failed as well.

In conclusion, humanized CD19 CAR-T cell therapy provided a treatment option for CD19-positive B-ALL patients who relapsed after or resisted prior murine CD19 CAR-T, hCD19 CAR-T followed by allo-transplantation allowed CR patients to obtain a longer event-free remission. Nevertheless, the prognosis of non-responders to hCD19 CAR-T remained dismal.

## Supplementary Information

**Additional file 1.**

## Data Availability

The datasets supporting the conclusions of this study are included in the article and supplemental data. For detailed and original data, please contact Dr. Lihong An at anlh@gobroadhealthcare.com, or corresponding author.

## References

[1] Maude SL, Frey N, Shaw PA, Aplenc R, Barrett DM, Bunin NJ (2014). Chimeric antigen receptor T cells for sustained remissions in leukemia. N Engl J Med.

[2] Davila ML, Riviere I, Wang X, Bartido S, Park J, Curran K (2014). Efficacy and toxicity management of 19-28z CAR T cell therapy in B cell acute lymphoblastic leukemia. Sci Transl Med.

[3] Maude SL, Laetsch TW, Buechner J, Rives S, Boyer M, Bittencourt H (2018). Tisagenlecleucel in children and young adults with B-cell lymphoblastic leukemia. N Engl J Med.

[4] Park JH, Rivière I, Gonen M, Wang X, Sénéchal B, Curran KJ (2018). Long-term follow-up of CD19 CAR therapy in acute lymphoblastic leukemia. N Engl J Med.

[5] Turtle CJ, Hanafi LA, Berger C, Gooley TA, Cherian S, Hudecek M (2016). CD19 CAR-T cells of defined CD4+: CD8+ composition in adult B cell ALL patients. J Clin Invest.

[6] Wagner DL, Fritsche E, Pulsipher MA, Ahmed N, Hamieh M, Hegde M (2021). Immunogenicity of CAR T cells in cancer therapy. Nat Rev Clin Oncol.

[7] Nie Y, Lu W, Chen D, Tu H, Guo Z, Zhou X (2020). Mechanisms underlying CD19-positive ALL relapse after anti-CD19 CAR T cell therapy and associated strategies. Biomark Res.

[8] Cao J, Wang G, Cheng H, Wei C, Qi K, Sang W (2018). Potent anti-leukemia activities of humanized CD19-targeted chimeric antigen receptor T (CAR-T) cells in patients with relapsed/refractory acute lymphoblastic leukemia. Am J Hematol.

[9] Zhao Y, Liu Z, Wang X, Wu H, Zhang J, Yang J (2019). Treatment with humanized selective CD19CAR-T cells shows efficacy in highly treated B-ALL patients who have relapsed after receiving murine-based CD19CAR-T therapies. Clin Cancer Res.

[10] Myers RM, Li Y, Barz Leahy A, Barrett DM, Teachey DT, Callahan C (2021). Humanized CD19-targeted chimeric antigen receptor (CAR) T cells in CAR-naive and CAR-exposed children and young adults with relapsed or refractory acute lymphoblastic leukemia. J Clin Oncol.

[11] Network NCC (2018). NCCN Clinical Practice Guidelines in Oncology: Acute Lymphoblastic Leukemia. Version 1.

[12] Lee DW, Santomasso BD, Locke FL, Ghobadi A, Turtle CJ, Brudno JN (2019). ASTCT consensus grading for cytokine release syndrome and neurologic toxicity associated with immune effector cells. Biol Blood Marrow Transplant..

[13] Liu S, Deng B, Yin Z, Lin Y, An L, Liu D (2021). Combination of CD19 and CD22 CAR-T cell therapy in relapsed B-cell acute lymphoblastic leukemia after allogeneic transplantation. Am J Hematol.

[14] Liu S, Deng B, Yin Z, Pan J, Lin Y, Ling Z (2020). Corticosteroids do not influence the efficacy and kinetics of CAR-T cells for B-cell acute lymphoblastic leukemia. Blood Cancer J.

[15] Schuster SJ, Svoboda J, Chong EA, Nasta SD, Mato AR, Anak Ö (2017). Chimeric antigen receptor T cells in refractory B-cell lymphomas. N Engl J Med.

[16] Greenbaum U, Mahadeo KM, Kebriaei P, Shpall EJ, Saini NY (2020). Chimeric antigen receptor T-cells in B-acute lymphoblastic leukemia: state of the art and future directions. Front Oncol.

[17] Wudhikarn K, Flynn JR, Rivière I, Gönen M, Wang X, Senechal B (2021). Interventions and outcomes of adult patients with B-ALL progressing after CD19 chimeric antigen receptor T-cell therapy. Blood..

[18] Doney K, Hagglund H, Leisenring W, Chauncey T, Appelbaum FR, Storb R (2003). Predictive factors for outcome of allogeneic hematopoietic cell transplantation for adult acute lymphoblastic leukemia. Biol Blood Marrow Transplant.

[19] Terwey TH, Hemmati PG, Martus P, Dietz E, Vuong LG, Massenkeil G (2010). A modified EBMT risk score and the hematopoietic cell transplantation-specific comorbidity index for pre-transplant risk assessment in adult acute lymphoblastic leukemia. Haematologica..

[20] Fry TJ, Shah NN, Orentas RJ, Stetler-Stevenson M, Yuan CM, Ramakrishna S (2018). CD22-targeted CAR T cells induce remission in B-ALL that is naive or resistant to CD19-targeted CAR immunotherapy. Nat Med.

[21] Pan J, Niu Q, Deng B, Liu S, Wu T, Gao Z (2019). CD22 CAR T-cell therapy in refractory or relapsed B acute lymphoblastic leukemia. Leukemia..

