# Supplementary material for: Humanized CD19 CAR-T cells in relapsed/refractory B-ALL patients who relapsed after or failed murine CD19 CAR-T therapy
Source: BMC Cancer. 2022 Apr 12;22:393. doi: 10.1186/s12885-022-09489-1 (PMC9004014; doi:10.1186/s12885-022-09489-1)
Supplement: Supplementary file 1 — Additional file 1. [file 12885_2022_9489_MOESM1_ESM.pdf]

Supplemental materials (combined PDF):

Supplemental methods
Supplemental figure legend
Figure S1. CAR-T cell expansion in peripheral blood (PB), detected by flow cytometry.
Table S1. Basic information of individual patients (n=19)
Table S2. hCD19 CAR-T treatment, response and follow-up
Table S3. Peak values of serum cytokines (pg/ml)

Supplemental Methods

Inclusion and exclusion criteria for enrollment

1. Inclusion criteria

1.1 Relapsed/refractory (r/r) B-cell acute lymphoblastic leukemia (B-ALL) patients who received prior murinized CD19 CAR (mCD19 CAR)-T cell therapy, the diagnostic standards for r/r B-ALL are according to NCCN guidelines (Version 1. 2018). Patients only with minimal residual disease (MRD) are not included. Extramedullary diseases (EMDs) are examined by PET-CT or CT/MRI/Ultrasonography for brain, chest, abdomen or local masses.

1.2 Patients must have a high level of CD19 antigen expression on leukemic blasts (⩾95% of blasts are positive for CD19), determined by multiparameter flow cytometry (FCM). For patients with isolated EMD without bone marrow (BM) involvement, blast cells are collected from fresh biopsy tissues or cerebrospinal fluid (CSF).

1.3 Age between 1 to 70 years old.

1.4 ECOG score 0-2.

1.5 The participant consent form must be signed, either by adult patients themselves or legal guardians of children under 18 years old.

2. Exclusion criteria

2.1 Severe organ dysfunction, such as acute heart failure or severe arrhythmia, dyspnea or acute respiratory failure, serum ALT/AST more than 10 times of normal range, serum creatinine/urea nitrogen over 2 times of normal range.

2.2 Obvious coagulopathy and bleeding.

2.3 Intracranial hypertension or unconscious, or with other central nervous diseases.

2.4 MRI or CT shows intracranial lesions.

2.5 Concomitant with other malignancy.

2.6 More than 20 leukemic cells/μl in cerebrospinal fluid (CSF) before CAR-T cell infusion.

2.7 More than 30% leukemic cells in peripheral blood (PB) before leukapheresis.

2.8 Sepsis or other uncontrolled infection.

2.9 In post-transplantation patients, ≥ grade II acute graft-versus-host disease (aGVHD) or ≥ moderate chronic GVHD (cGVHD).

2.10 Active viral infection, including hepatitis B, hepatitis C, HIV, CMV, EBV.

2.11 Pregnant or breastfeeding women.

2.12 Other situations may interfere the treatment effect or increase the risk of candidates.

Supplemental figure legend

Figure S1. CAR-T cell expansion in peripheral blood (PB), detected by flow cytometry. CAR-T cell expansion was seen in 17 patients, the median peak number of CAR-T cells in PB was 5.2 x10^6/L. CAR-T cells could not be detected in 9 patients (53%, 9/17) within 10-37 days after cell infusion. For another 8 patients, 5 immediately underwent HCT after CAR-T and no longer did CAR-T cell detection; 2 had not come back since discharge from our hospital (1 with no response died and 1 CR patient underwent HCT in local hospital); only 1 case had follow-up data, her CAR-T cells were not detectable in PB on day 68, but were detectable in bone marrow till day 153 (0.6%-2.38% in lymphocytes), she refused transplantation and relapsed at 9 months after hCD19 CAR-T.

Figure S1

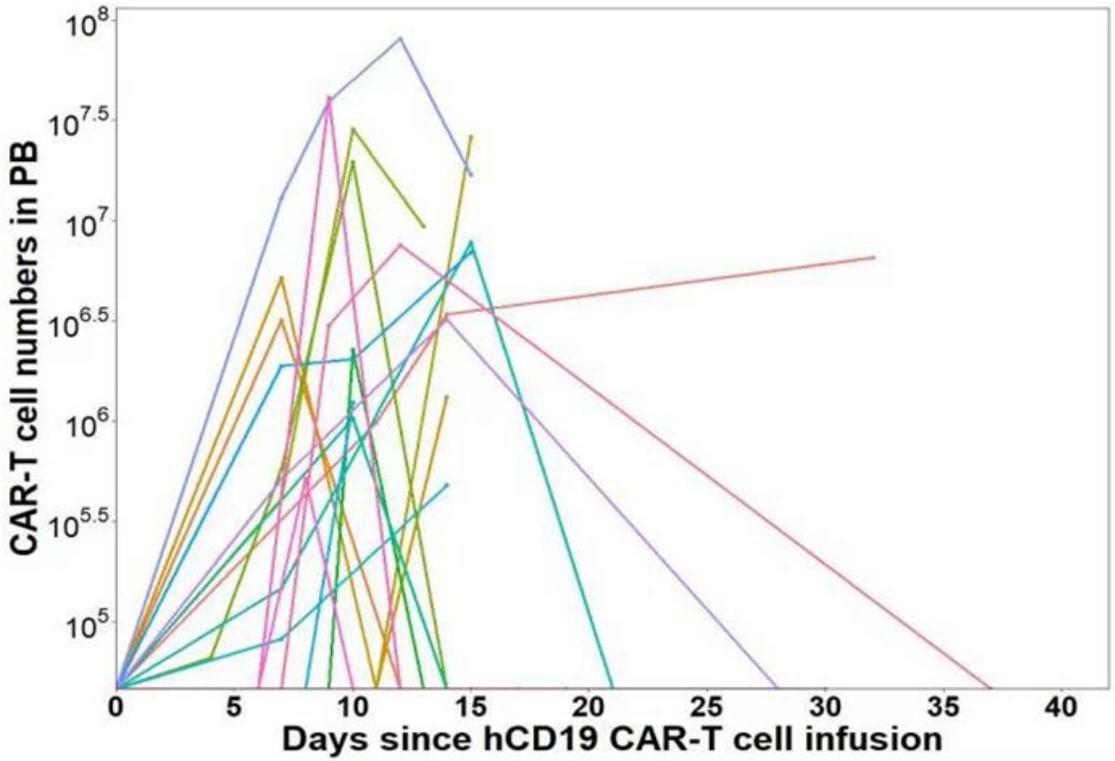

10^8
$10^{7.5}$
10^7
$10^{6.5}$
10^6
$10^{5.5}$
10^5
CAR-T cell numbers in PB
0
5
10
15
20
25
30
35
40
Days since hCD19 CAR-T cell infusion

Table S1. Basic information of individual patients (n=19)

Pt.No.	Age	Sex	Tumor burden at enrollment		Adverse genetic change	Prior allo-HCT	Previous mCD19 CAR-T		Interval time between mCD19 and hCD19 CAR-T (months)
			Blasts in BM (%)[a]	EMD[b]			Number	Response	
1	13	F	48.5	–	–	No	1	NR	0.9
2	4	F	57.5	–	TP53 and KRAS gene mutations	No	2	CR/NR	First, 4.8/second, 1.6
3	49	F	43	–	BCR/ABL	Yes	1	CR	29.6
4	6	F	7	–	PTPN11 gene mutation	Yes	1	CR	10.4
5	38	F	0.11	Multiple sites (including CNS)	–	Yes	1	CR	20
6	47	M	33.5	–		Yes	1	CR	19
7	18	M	32.5	–	BCR/ABL	Yes	1	CR	9
8	31	F	51	Multiple sites	–	Yes	1	PR	16.3
9	15	M	1.76	CNS	–	Yes	1	CR	55
10	7	M	46	–	KRAS and PTPN11 gene mutation	No	1	CR	16.6
11	47	M	0.02	Multiple sites	–	Yes	1	CR	5
12	20	M	0	Multiple sites	IKZF1 deletion	Yes	1	CR	26.7
13	9	F	81	CNS	–	No	1	CR	20.8
14	29	M	98	–	–	No	1 (with CD22)	CR	8.7

15	21	M	74.5	–	CRLF2 rearrangement and NRAS gene mutation	No	2 (both with CD22)	CR/CR	First, 17.3/second, 5
16	17	M	98	–	TP53, KRAS, PTPN11 and NF1 gene mutations	No	1	NR	1.5
17	34	F	1.86	Multiple sites	–	Yes	1	MRD+ CR	8.4
18	5	F	95	–	–	No	2 (second time with CD22)	NR/NR	First, 2/second, 0.9
19	22	M	86	–	EBF1-PDGFRB fusion gene, IKZF deletion	No	1	CR	27.5

[a]Blasts were determined by flow cytometry when morphologic count was<5%. [b]Multiple sites were defined as ≥2 sites of EMD, including central nervous system, lymph nodes, bones, soft masses, mediastinum, nasopharynx and testis.

Abbreviations: Pt.No., patient number; BM, bone marrow; EMD, extramedullary disease; allo-HCT, allogeneic hematopoietic cell transplantation; mCD19, murinized CD19; hCD19, humanized CD19; NR, no response; CR, complete remission, including CR with incomplete blood count recovery; PR, partial remission; CNS, central nervous system.

Table S2. hCD19 CAR-T treatment, response and follow-up

Pt. No.	Dosage of infused cells ($\times 10^5$/kg)	Peak CAR-T cell numbers in PB ($\times 10^6$/L)	CRS grade	Neuro-toxicity	Treatment response on day 30	Follow-up	
						Allo-HCT	Non-HCT
1	6.4	6.51	1		CR	relapsed and died	
2	5	1.24	1		CR	CR	
3	1	6.88	2		CR	2nd HCT, CR	
4	2	80.1	1		NR	2nd HCT, relapsed and survive	
5	2	3.22	2	1	CRi	2nd HCT, CR	
6	2	0	0		NR	2nd HCT, relapsed and died	
7	1.95	0.52	1		NR		disease progressed and died
8	1	40.7	1		NR		disease progressed and died
9	5	7.53	1		CR		relapsed and died
10	18	3.19	1		CR	CR	
11	1	5.18	1		NR		disease progressed and died
12	5	26	1		NR	2nd HCT, CR	
13	5	28.5	1		CRi		relapsed and survive
14	3	19.6	1		CR	CR	
15	2.17	2.26	4		CRi	CR	
16	2.44	0	1		CRi	CR	
17	2.96	1.03	1		CR	2nd HCT, CR	
18	2.07	7.75	2	1	CRi	CR	
19	5	0.48	1		CR	CR	

Abbreviations: Pt.No., patient number; PB, peripheral blood; CRS, cytokine release syndrome; allo-HCT, allogeneic hematopoietic cell transplantation; CR, complete remission; CRi, CR with incomplete blood count recovery; NR, no response.

Table S3. Peak values of serum cytokines (pg/ml)

Pt.No	IL-6	TNF-α	IL-10	sCD25	IFN-γ
1	13.54	16.85	9.04	812	
2	53.09	16.77	62.7	2816	51.79
3	17.92	25.94	46.34	4232	382.86
4	9.14	12.26	171.26	3040	22.51
5	92.19	6.14	8.03	5004	5.1
6	28.34	18.58	10.4	2252	8.52
7	225.8	59.36	169.68	12984	130.89
8	1240	128.18	621.05	11208	24.03
9	107.4	20.6	67.12	8364	135.41
10	565.7	60.2	291.35	9916	
11	371.7	18.49	98.15	2414	8.14
12	18.01	6.5	18.02	3680	40.98
13	67.54	5.56	101.03	6964	56.1
14	2588.1	52.54	710.13	38716	2008.34
15	163.1	66.06	626.45	9500	1650.06
16	320	88.52	530.94	10930	1751.85
17	2.13	19.48	13.01	3047	24.36
18	110.3	9.56	24.89	20726	77.54
19	60.62	27.54	303.02	10439	66.64
